# Detection of anti*-Trypanosoma* spp. antibodies in cattle from southern Brazil

**DOI:** 10.1590/S1984-29612024002

**Published:** 2023-12-22

**Authors:** Gisele Vaz Aguirre Samoel, Fagner D’ambroso Fernandes, Isac Junior Roman, Bibiana Teixeira Rodrigues, Luiz Claudio Miletti, Patrícia Bräunig, Renata Rojas Guerra, Luís Antônio Sangioni, Juliana Felipetto Cargnelutti, Fernanda Silveira Flores Vogel

**Affiliations:** 1 Laboratório de Doenças Parasitárias, Departamento de Medicina Veterinária Preventiva, Universidade Federal de Santa Maria – UFSM, Santa Maria, RS, Brasil; 2 Centro Universitário Ritter dos Reis – UniRitter, Porto Alegre, RS, Brasil; 3 Universidade do Estado de Santa Catarina – UDESC, Florianópolis, SC, Brasil; 4 Departamento de Estatística, Universidade Federal de Santa Maria – UFSM, Santa Maria, RS, Brasil; 5 Laboratório de Bacteriologia, Departamento de Medicina Veterinária Preventiva, Universidade Federal de Santa Maria – UFSM, Santa Maria, RS, Brasil

**Keywords:** Trypanosoma vivax, serological diagnosis, epidemiology, Trypanosoma vivax, diagnóstico sorológico, epidemiologia

## Abstract

Bovine trypanosomosis, caused by *Trypanosoma vivax*, is a disease that originated in Africa and currently affects cattle in several South American countries, including almost all Brazilian states. Despite the reports on *T. vivax* infection in southern Brazil, data on its circulation status is currently unavailable. In this study, we aimed to detect anti-*Trypanosoma* spp. IgG antibodies in cattle from Rio Grande do Sul and suggest areas with *T. vivax* transmission risk. A total of 691 serum samples from cattle in the intermediate regions of Rio Grande do Sul were analyzed using indirect immunofluorescence assay (IFA). The overall seroprevalence of anti-*Trypanosoma* antibodies in cattle was 24.6% (170/691). The detection rate ranged from 0-37.3%, with a high prevalence in the intermediate regions of Ijuí (37.3%), Uruguaiana (30.7%), and Passo Fundo (28.9%). Thus, these regions were suggested as possible bovine trypanosomosis risk areas due to the high seroprevalence. This is the first serological study to determine *Trypanosoma* spp. infection status in cattle from Rio Grande do Sul, providing data on the epidemiology of trypanosomosis in the state.

## Resumo

A tripanossomose bovina, causada por *Trypanosoma vivax,* é uma doença que teve origem na África, e atualmente, afeta bovinos em diversos países da América do Sul, incluindo quase todos os estados brasileiros. Apesar dos relatos de infecção por *T. vivax*, no sul do Brasil, dados sobre seu status de circulação não estão disponíveis atualmente. Neste estudo, o objetivo foi detectar anticorpos IgG anti-*Trypanosoma* spp. em bovinos do Rio Grande do Sul e sugerir possíveis áreas de risco de transmissão de *T. vivax*. Foi analisado um total de 691 amostras de soro de bovinos, das regiões intermediárias do Rio Grande do Sul, usando-se o ensaio de imunofluorescência indireta (IFA). A soroprevalência geral de anticorpos anti-*Trypanosoma* em bovinos foi de 24,6% (170/691). A taxa de detecção variou de 0 a 37,3%, com uma alta prevalência nas regiões intermediárias de Ijuí (37,3%), Uruguaiana (30,7%) e Passo Fundo (28,9%). Portanto, essas regiões foram sugeridas como possíveis áreas de risco para a tripanossomose bovina, devido à alta soroprevalência. Este é o primeiro estudo sorológico a determinar o status de infecção por *Trypanosoma* spp., em bovinos do Rio Grande do Sul, fornecendo dados sobre a epidemiologia da tripanossomose no estado.

## Introduction

Trypanosomes are flagellate protozoans belonging to the family Trypanosomatidae and genus *Trypanosoma*. Various parasitical species belonging to *Trypanosoma*, such as *Trypanosoma brucei*, *Trypanosoma congolense*, *Trypanosoma evansi*, and *Trypanosoma vivax*, cause trypanosomosis, a livestock disease prevalent in Africa, Latin America, and Asia (Stevens & Brisse, 2004). In South America, *T. vivax* is the most important causative agent of trypanosomosis in cattle, causing losses in the cattle industry. Other species, such as non-pathogenic *T. theileri* and *T. evansi*, also infect cattle but rarely cause diseases (Radostitis et al., 2007). Economic losses in Brazilian cattle-rearing are associated with animal mortality following outbreaks, and indirect interferences of subclinical and undiagnosed infections that reduce weight gain and milk production as well as cause abortion, infertility, and other reproductive disorders, thus implicating livestock productivity (Otte et al., 1994; Seidl et al., 1999; Jones & Dávila, 2001).

*T. vivax* was introduced in Latin America through infected cattle imported from Africa and has spread across several countries (Jones & Dávila, 2001). *T. vivax*-mediated bovine trypanosomosis was first reported in the Amazon region of Brazil: in buffalos from Pará (Shaw & Lainson, 1972) and cattle from Amapá (Serra-Freire, 1981). Subsequently, infections in cattle herds occurred with increasing frequency, with reported *T. vivax* infection in other states in the North (Linhares et al., 2006), Northeast (Batista et al., 2007; Guerra et al., 2013; Lopes et al., 2018), Midwest (Silva et al., 1996; Osório et al., 2008), Southeast (Carvalho et al., 2012; Cadioli et al., 2012), and South Brazil (Silva et al., 2009). Currently, *T. vivax* is considered endemic in some Pantanal and Amazon rainforest regions.

African dissemination of *T. vivax* includes cyclical transmission by *Glossina* spp., with *T. vivax* developing in their digestive tract. Since *Glossina* spp. is absent in South America, the major transmission methods include non-cyclical or mechanical transmission by blood-sucking flies (Otte & Abuabara, 1991), such as tabanids (horseflies) and *Stomoxys calcitrans* (stable flies). Although not confirmed, cyclical transmission may involve one or more vector species (Otte & Abuabara, 1991). Furthermore, iatrogenic transmission through fomites and transplacental infections have also been described (Cadioli et al., 2012). *T. vivax* infects various domestic and wild species, including sheep, goats, horses, and cervids, which can act as important reservoirs (Dávila et al., 2003).

In South America, *T. vivax* infection manifests variable virulence and pathogenicity levels (Gardiner & Mahmoud, 1992), causing non-specific clinical signs, such as severe anemia, weight loss, edema, immunosuppression, and reproductive failure. Some acute cases can develop various neurological disorders that eventually cause the death of the affected animals (Gonzatti et al., 2014). Asymptomatic and chronic infections are common in cattle from endemic regions and can be reactivated by nutritional and physical stress, concomitant disease, pregnancy, and lactation (Garcia et al., 2016). No single clinical sign is pathognomonic and the disease may simulate many other infections (Büscher, 2014); therefore, it is easily overlooked or confused with other diseases.

In southern Brazil, despite reports of *T. vivax* infection in cattle (Silva et al., 2009) and naturally infected horses (Silva et al., 2011), no data on pathogen circulation status in Rio Grande do Sul (RS) have been recorded. RS is geographically located between territories that have characteristics that may favor the spread of the disease. The region of Argentina, which borders Brazil, has numerous cattle herds and the culture of practicing rodeos, in addition, there are several reports of animal trafficking to Brazil. This can provide a risk factor for the introduction of diseases to brazilian heards. Thus, understanding the actual *T. vivax* circulation in RS is important. This study aimed to detect anti-*Trypanosoma* spp. antibodies in cattle in the regions of RS and elucidate the trypanosomosis transmission risk areas in the region under study.

## Materials and Methods

A total of 691 serum samples were obtained from the sera bank of Laboratório de Doenças Parasitárias of Universidade Federal de Santa Maria (LADOPAR/UFSM) from 2020-2022. Samples were from taurine (*Bos taurus*), zebu (*Bos indicus*), and crossbreed cattle in the intermediate regions of RS: Uruguaiana, Santa Maria, Porto Alegre, Passo Fundo, Ijuí, Caxias do Sul, Santa Cruz do Sul, and Pelotas (IBGE, 2017). The total number of samples was determined based on statistical analysis using the Epi Info (v7.2.5) system according to the cattle population of RS (IBGE, 2022). The confidence interval was 95%, with 3% standard error.

Indirect immunofluorescence assay (IFA) described by Camargo (1966) was used to detect IgG antibodies against *Trypanosoma* spp. with some modifications. Briefly, IFA was performed using a microscopic strain infected with *T. vivax* trypomastigotes. The serum samples were diluted in phosphate buffered saline (pH 7.2) to 1:80 (Cuglovici et al., 2010). Commercial fluorescein-labeled anti-bovine IgG^©^ (Rabbit Anti-Bovine IgG FITC®, F7884, Sigma Bio Sciences, St. Louis, Missouri, USA) secondary antibody was diluted to 1:400. The samples were incubated with both the primary and secondary antibodies for 50 min at 37 °C in a dark and humid chamber. Confirmed positive and negative serum samples diluted to 1:80 were used as controls. The slides were observed at 400× magnification under a fluorescence microscope (Optiphase INV403F). The samples were considered reactive when the trypomastigotes revealed total fluorescence, showing binding between the FITC-labeled secondary antibody and the antigen–antibody complex. Serum that did not show fluorescence was considered non-reactive.

We conducted an exploratory data analysis of the samples. The investigation involved calculating the absolute frequencies and prevalence of cattle trypanosomosis. Additionally, contingency tables were constructed, considering each herd's intermediate region and the cattle breeding. To assess the presence of significant differences in the prevalence of these variables, we performed the Fisher’s exact test, considering 5% as the significance level.

## Results

Anti-*Trypanosoma* spp. IgG was detected in 24.6% (170/691) cattle serum samples. The detection rate in the study region was 0-63.6% (Table 1). A total of 24 herds were sampled, of which 9 and 5 were dairy cattle and beef cattle, respectively, and 10 herds did not report this information. At least one positive sample was detected in 21 herds (89.6%). The detection frequency in the positive herds ranged from 4.2-63.6%. The highest detection frequencies were observed in three dairy herds from Passo Fundo (63.6%, 7/11; 46.1%, 6/13; and 45.4%, 10/22), one herd from Porto Alegre (39.1%, 9/23), and one herd from Pelotas (39.3%, 22/56) (Table 1).

**Table 1:** Absolute frequencies and prevalences by herd for the serological detection of *Trypanosoma* spp. in cattle serum samples collected from the Rio Grande do Sul intermediate regions.

**Herd Identifier**	**Trypanosomosis**		**Total**	**Prevalence (%)**	**Intermediate Region**	**Cattle breeding**
	**Positive**	**Negative**				
1	0	19	19	0.0	Caxias do Sul	Not informed
2	0	7	7	0.0	Ijuí	Not informed
3	0	7	7	0.0	Ijuí	Not informed
4	31	38	69	44.9	Ijuí	Dairy Cattle
5	4	35	39	10.3	Passo Fundo	Dairy Cattle
6	6	7	13	46.1	Passo Fundo	Dairy Cattle
7	7	4	11	63.6	Passo Fundo	Dairy Cattle
8	4	9	13	30.8	Passo Fundo	Dairy Cattle
9	10	12	22	45.5	Passo Fundo	Dairy Cattle
10	9	31	40	22.5	Passo Fundo	Dairy Cattle
11	22	34	56	39.3	Pelotas	Not informed
12	1	23	24	4.2	Pelotas	Not informed
13	3	24	27	11.1	Pelotas	Not informed
14	9	14	23	39.1	Porto Alegre	Not informed
15	5	52	57	8.8	Porto Alegre	Not informed
16	5	14	19	26.3	Santa Cruz do Sul	Dairy Cattle
17	2	16	18	11.1	Santa Cruz do Sul	Not informed
18	2	6	8	25.0	Santa Cruz do Sul	Not informed
19	4	65	69	5.8	Santa Maria	Dairy Cattle
20	3	9	12	25.0	Uruguaiana	Beef Cattle
21	3	5	8	37.5	Uruguaiana	Beef Cattle
22	3	16	19	15.8	Uruguaiana	Beef Cattle
23	1	8	9	11.1	Uruguaiana	Beef Cattle
24	36	66	102	35.3	Uruguaiana	Beef Cattle
Total	170	521	691	24.6		

**Figure 1:**
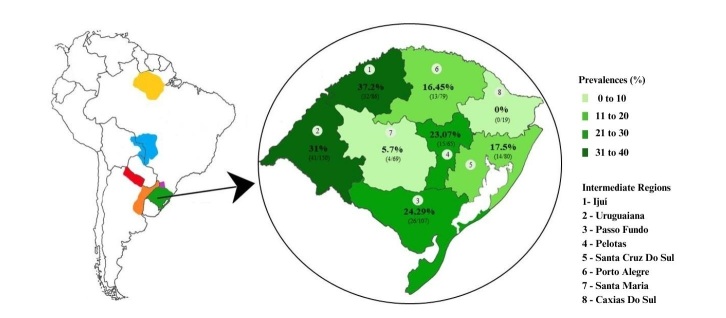
Map of Rio Grande do Sul (right) showing *Trypanosoma* spp. seroprevalence in cattle according to the intermediate regions. (Left) Map showing *T. vivax* occurrence in South America: upper Rio Paraguay basin, including portions of the Brazilian, Bolivian, and Paraguayan Pantanal regions (blue); Alto Paraná, Amambay, Canindeyú, and Concepción regions of Paraguay (pink); western Santa Catarina (purple); Formosa region of Argentina (red); Mesopotamia Argentina (orange); and Rio Grande do Sul (green).

Regarding the intermediate regions, the higher prevalences were found in Ijuí (37.3%), Uruguaiana (30.7%), and Passo Fundo (28.9%), respectively (Table 2 and Figure 1). The lowest prevalence was observed in Pelotas (24.3%), Santa Cruz do Sul (23.0%), Porto Alegre (17.5%), Santa Maria (5.7%), and Caxias do Sul (0%) (Table 2 and Figure 1). When calculating prevalence ratio using Santa Maria as reference, we verify that the prevalence is 6.4 times higher in Ijuí than in herds from this region. In addition, besides Caxias do Sul, which did not present any prevalent case, all the regions showed a prevalence more than three times higher than Santa Maria. The Fisher's exact test corroborates this result since it returns a p-value smaller than 0.001, leading to the rejection of the hypothesis that the prevalences are equal in all intermediate regions at the significance level of 5%. The Cattle breeding comparison also leads to significant differences (p-value=0.006 in the Fisher's exact test). It means that bovine trypanosomiasis differs within the categories of cattle breeding. The prevalence ratio shows that bovine trypanosomiasis in beef cattle is 1.7 times higher than in cattle with unspecified breeding. For dairy cattle, the prevalence ratio indicates that its prevalence is 1.5 times higher than in cattle with unspecified breeding.

**Table 2:** Contingency tables and prevalences by intermediate region and cattle breeding.

**Variable**	**Trypanosomosis**		**Total**	**Prevalence (%)**	**Prevalence ratio**
	**Positive**	**Negative**			
**Intermediate Region**					
Caxias Do Sul	0	19	19	0.0	0.0
Ijuí	31	52	83	37.3	6.4
Passo Fundo	40	98	138	29.0	5.0
Pelotas	26	81	107	24.3	4.2
Porto Alegre	14	66	80	17.5	3.0
Santa Cruz Do Sul	9	36	45	20.0	3.4
Santa Maria	4	65	69	5.8	1.0
Uruguaiana	46	104	150	30.7	5.3
**Cattle breeding**					
Beef Cattle	46	104	150	30.7	1.7
Dairy Cattle	80	215	295	27.1	1.2
Not informed	44	202	246	17.9	1.0

## Discussion

Trypanosomosis, caused by *T. vivax*, is considered a non-endemic disease in cattle in southern Brazil. Currently, this species is only considered endemic in some Pantanal and Amazon rainforest regions. However, it sporadically infects various species throughout southern Brazil regions. *T. vivax* infects cattle and horses with clinical signs in the central cities of RS (Silva et al., 2009, 2011). Nevertheless, since serological studies have not been performed using serum samples collected in RS, no data on cattle trypanosomosis in RS is available. This is the first bovine serological study on anti-*Trypanosoma* spp. antibody detection and frequency among all regions of RS, highlighting the importance of widespread disease-causing pathogen analysis for the differential diagnosis of agents that cause hemolytic, neurological, or reproductive disorders.

Though RS is not a *T. vivax* endemic region, the presence of anti-*Trypanosoma* spp. antibodies over almost all intermediate regions (7/8) indicated that trypanosomes have spread throughout the cattle from RS and may be associated with symptomatic or asymptomatic infections in beef and dairy herds. According to these findings, there is a risk of trypanosomosis outbreaks in the state, mainly in the cities of intermediate regions with high seroprevalence detection. The presence of vectors and favorable conditions for their development (mainly *S. calcitrans*), food restriction, inadequate transport conditions, and thermal stress in dairy cattle herds is associated with the needle-sharing practice during oxytocin application in lactating cows; these factors, among other, favor disease epidemiology in cattle herds in Brazil (Cadioli et al., 2012).

The detection rate in RS (24.6%) is lower than that in endemic areas such as the Pantanal and Amazon rainforests (93.1-98.5%; Guedes et al., 2008; De Mello et al., 2019), but higher than that in enzootic unstable areas for *T. vivax* (11.90-15.99%; Guerra et al., 2013). However, the seroprevalence results can vary according to the study region, herd, seasonality, and cattle breeding management type.

The difference in the prevalence of antibodies against anti-*Trypanosoma* spp. in different regions in RS support defining areas for trypanosomosis transmission risk. The western intermediate regions of Uruguaiana and Ijuí had the highest serological prevalence, with over 30% detection frequency.

Several circumstances contribute to trypanosome dissemination over a region. The western RS is characterized by a high cattle population with proprieties that have animal trafficking characteristics. The national and international live livestock trade represents a serious risk factor; moreover, the introduction of animals infected with subclinical or chronic trypanosomosis is the main risk factor for *T. vivax* dissemination from endemic to non-endemic regions. Further studies on naturally infected herds have demonstrated that *T. vivax* induces chronic asymptomatic infection in cattle from endemic areas (Ventura et al., 2001; Dávila et al., 2003). Another concerning observation in animal trade is the occurrence of horses as *T. vivax* reservoirs, as observed in Paraguayan regions that border southwest Brazil and Argentina (Suganuma et al., 2022). In both animal species, obligatory *T. vivax*-mediated trypanosomosis diagnosis is not required for international or national trade.

Territorial proximity facilitates pathogen dispersion from different locations, which is an epidemiologically important factor because western RS borders Santa Catarina state and Argentina, where *T. vivax* was detected in cattle (Paoletta et al., 2018; Silva et al., 2022). Some serological studies using symptomatic cattle from the western region of Santa Catarina detected 39.0% (57/146) seropositive animals, suggesting *T. vivax* existence in these areas. In the Argentine territory, *T. vivax* outbreaks have been described in cattle from Formosa and Mesopotamia Argentina. The agro-ecological conditions of these neighboring regions are similar to those of western RS, with potential risk of dispersion among the Pampa biome regions that border Brazil (Abdala et al., 2021; Monzon et al., 2013; Paoletta et al., 2018). Moreover, the main intermediate regions (Ijuí, Uruguaiana, and Passo Fundo) where anti-*Trypanosoma* spp. antibodies were detected in this study, border these locations in Santa Catarina and Argentina. Thus, it is important to suggest the intermediate regions of Ijuí, Uruguaiana, and Passo Fundo as risk areas for trypanosomosis in cattle from RS.

Immunological cross-reactivity between trypanosome species may occur, mainly between *T. vivax* and *T. evansi*, which share many common antigens (Uzcanga et al., 2004). Their coexistence in southern Brazil makes precise interpretation of serological tests difficult, necessitating the use of molecular tools to determine the species involved. Furthermore, the new species discovered in Argentina may also be present in Brazilian herds and must be considered without knowing its influence on immunological tests. Therefore, antibodies should be associated with specific species with caution; the antibodies found in this study are not considered *T. vivax*-specific.

Despite the high detection rate in cattle from the same herd, anti-*Trypanosoma* spp. IgG antibodies were observed in many dairy herds from Passo Fundo, Porto Alegre, and Pelotas. Rainy periods and/or high humidity contribute to developing mechanical vectors in the environment, as well as improving the possibility of increasing the infection rate of cattle from the same herd. This phenomenon was also observed by Cuglovici et al. (2010), where seroprevalence ratios in dairy cattle herds from Igarapé and Minas Gerais were 7.4-48.0%, with the highest incidence was correlated with an increased *S. calcitrans* population during rainy seasons. Thereby, *S. calcitrans* can corroborate trypanosomosis epidemiology in RS, as it is a widely distributed ectoparasite in RS.

In summary, detecting antibodies against *Trypanosoma* spp. in cattle from RS provides evidence that these herds have been exposed to these pathogens. Further studies on trypanosomosis epidemiology should attempt to conclusively link the pathogens to animal production losses. Owing to the possibility of cross-reactions with other trypanosomes in serological methods, combined parasitological and molecular methods would provide a clear understanding of the specific species infecting the seropositive animals and the epizootiological situation of the trypanosomes present in cattle herds from RS.

## Conclusion

This is the first serological study of trypanosomosis in cattle from RS. This study detected antibodies against *Trypanosoma* spp. in cattle from almost all intermediate regions, thus suggesting a possible risk of trypanosomosis outbreaks, mainly in animals from the western RS. The intermediate regions from Ijuí, Uruguaiana, and Passo Fundo were suggested as risk areas for bovine trypanosomosis due to high seroprevalence. These findings highlight the need for an effective surveillance program to diagnose and prevent trypanosomosis spread, thus reducing disease impact on livestock productivity, which may otherwise be overlooked and underdiagnosed in RS.
